# Preclinical and clinical characterization of the RORγt inhibitor JNJ-61803534

**DOI:** 10.1038/s41598-021-90497-9

**Published:** 2021-05-26

**Authors:** Xiaohua Xue, Aimee De Leon-Tabaldo, Rosa Luna-Roman, Glenda Castro, Michael Albers, Freddy Schoetens, Samuel DePrimo, Damayanthi Devineni, Thomas Wilde, Steve Goldberg, Olaf Kinzel, Thomas Hoffmann, Anne M. Fourie, Robin L. Thurmond

**Affiliations:** 1 Janssen Research & Development, LLC, La Jolla, CA USA; 2 Janssen Research & Development, LLC, Spring House, PA USA; 3 Department of Research, Phenex Pharmaceuticals AG, Heidelberg, Germany

**Keywords:** Drug discovery, Immunology

## Abstract

The nuclear receptor retinoid-related orphan receptor gamma t (RORγt) plays a critical role in driving Th17 cell differentiation and expansion, as well as IL-17 production in innate and adaptive immune cells. The IL-23/IL-17 axis is implicated in several autoimmune and inflammatory diseases, and biologics targeting IL-23 and IL-17 have shown significant clinical efficacy in treating psoriasis and psoriatic arthritis. JNJ-61803534 is a potent RORγt inverse agonist, selectively inhibiting RORγt-driven transcription versus closely-related family members, RORα and RORβ. JNJ-61803534 inhibited IL-17A production in human CD4^+^ T cells under Th17 differentiation conditions, but did not inhibit IFNγ production under Th1 differentiation conditions, and had no impact on in vitro differentiation of regulatory T cells (Treg), nor on the suppressive activity of natural Tregs. In the mouse collagen-induced arthritis model, JNJ-61803534 dose-dependently attenuated inflammation, achieving ~ 90% maximum inhibition of clinical score. JNJ-61803534 significantly inhibited disease score in the imiquimod-induced mouse skin inflammation model, and dose-dependently inhibited the expression of RORγt-regulated genes, including IL-17A, IL-17F, IL-22 and IL-23R. Preclinical 1-month toxicity studies in rats and dogs identified doses that were well tolerated supporting progression into first-in-human studies. An oral formulation of JNJ-61803534 was studied in a phase 1 randomized double-blind study in healthy human volunteers to assess safety, pharmacokinetics, and pharmacodynamics. The compound was well tolerated in single ascending doses (SAD) up to 200 mg, and exhibited dose-dependent increases in exposure upon oral dosing, with a plasma half-life of 164 to 170 h. In addition, dose-dependent inhibition of ex vivo stimulated IL-17A production in whole blood was observed, demonstrating in vivo target engagement. In conclusion, JNJ-61803534 is a potent and selective RORγt inhibitor that exhibited acceptable preclinical safety and efficacy, as well as an acceptable safety profile in a healthy volunteer SAD study, with clear evidence of a pharmacodynamic effect in humans.

## Introduction

The retinoic acid receptor-related (ROR) sub-family of orphan nuclear receptors (reviewed in^1^) consists of isoforms of RORα, β and γ generated from their corresponding genes through alternative promoter usage and exon splicing. These isoforms exhibit differential tissue expression and functions. RORγt is a differentially spliced variant of RORγ, that differs only in the N-terminus by the presence of 21 additional amino acids in RORγ. The specific endogenous physiological ligand for RORγt/RORγ remains unclear but a few have been reported including 7β-27-dihydroxy cholesterol^2^, two other cholesterol biosynthetic intermediates^3,4^, and endogenously produced vitamin D and lumisterol hydroxyderivatives^5,6^.

RORγt is exclusively expressed in immune cells including CD4^+^CD8^+^double positive thymocytes^7^, Th17^8^, Tc17^9^, regulatory T cells (Tregs)^10,11^, invariant natural killer T (iNKT)^12^, γδ T cells^13^, NK cells^14^, and a subset of innate lymphoid cells (ILCs)^15^. RORγt is a key transcription factor regulating Th17 cell differentiation and expansion, and driving the expression of IL-23 receptor and production of IL-17A, IL-17F and IL-22 in innate and adaptive immune cells, also termed “type 17” cells^16^. Cytokines such as IL-17A, IL-17F, and IL-22 bind to their receptors on tissue cells inducing the production of various inflammatory chemokines, cytokines and metalloproteases, resulting in activation and recruitment of immune cells to the site of injury or inflammation, which maintain and amplify the proinflammatory response^17^. The Th17 cell subset has been shown to be the major pathogenic population in several models of autoimmune inflammation, including collagen-induced arthritis (CIA), experimental autoimmune encephalomyelitis (EAE)^18,19^, and non-alcoholic steatohepatitis (NASH)^20^. Transgenic mice overexpressing RORγt in T cells become susceptible to Theiler’s murine encephalomyelitis virus-induced demyelinating disease, a viral model for multiple sclerosis^21^. RORγt-deficient mice show decreased susceptibility to EAE^8^ and skin inflammation^22^. RORγt-deficient T cells fail to induce colitis in the mouse T cell transfer model^23^.

In human genetic studies, polymorphisms in the genes for Th17 cell-surface receptors, IL-23R and CCR6, have been found to be associated with susceptibility to inflammatory bowel disease, multiple sclerosis, rheumatoid arthritis, ankylosing spondylitis and psoriasis^24–29^. Therapeutic intervention with biologics targeting IL-12/23, IL-23, IL-17A or IL-17RA has provided clinical validation for the critical role of IL-23/IL-17 pathway in human autoimmune diseases^30–36^. RORγt is a master regulator lying at the core of this pathway, representing a novel opportunity for immune-mediated disease intervention. Studies have shown that RORγt is tractable to modulation by oral small molecules^37–39^.

We describe here a novel, selective and potent RORγt inverse agonist, JNJ-61803534. This molecule specifically blocked RORγt-dependent pathways in cellular assays and significantly reduced inflammation in preclinical models. GLP toxicology studies supported clinical testing and a single ascending dose phase 1 clinical study demonstrated an acceptable clinical safety profile, and correlation of pharmacokinetics and pharmacodynamics.

## Results

### In vitro pharmacology

**Figure 1:**
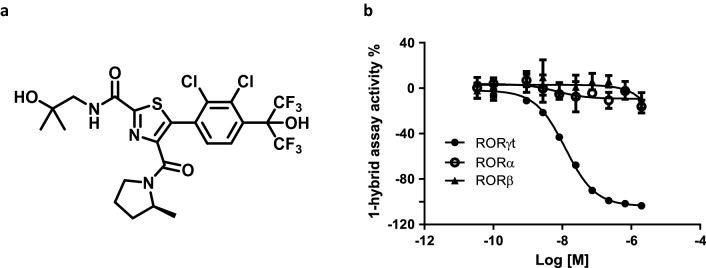
Structure and selectivity of JNJ-61803534 for inhibition of RORγt-driven transcription. **(a)** Structure of JNJ-61803534. **(b)** Activity of JNJ-61803534 in 1-hybrid reporter assays. HEK-293 T cells were transfected with vectors encoding RORγt, RORα or RORβ, respectively, fused with the GAL4 DNA binding domain. After incubation with compound overnight, luciferase signals were measured. JNJ-61803534 was tested at a starting concentration of 2 μM in three-fold serial dilutions in duplicate.

Through high-throughput screening and structure–activity relationship development, several chemotypes were identified that bound to the RORγt ligand binding domain, and demonstrated dose-dependent functional inhibition of RORγt in cell-based reporter assays^40–45^. JNJ-61803534 (US10,150,762 B2) was developed through optimization of a thiazole series^41,44^ and the chemical structure is shown in Fig. 1a. In the 1-hybrid reporter assay, JNJ-61803534 showed potent, dose-dependent inhibition of RORγt-driven transcription, with an IC_50_ of 9.6 ± 6 nM. In comparison, IC_50_ values for RORα and RORβ were > 2 µM in similar assays (Fig. 1b), demonstrating high selectivity for RORγt.

To evaluate its selectivity, JNJ-61803534 was tested against 18 human nuclear receptors including TRα, RARα, PPARα, PPARβ, PPARγ, LXRβ, FXR, VDR, PXR, CAR, RXRα, ERα, ERβ, GR, MR, PR and AR in a cellular GAL4 reporter assay, and ERRγ in a biochemical TR-FRET assay, in agonist and antagonist mode and showed 35-fold selectivity over PXR and > 167-fold over the other nuclear receptors tested. In addition, JNJ-61803534 was evaluated for its activity against a panel of 52 receptors, ion channels and transporters, 28 GPCRs in agonist and antagonist mode, and 46 kinases, and did not demonstrate significant binding or functional activity except against adenosine A3 receptor, NK2, Na^+^-channel and Cl- channel which were inhibited at > 50% at 10 µM. However, in follow-up functional studies, the IC_50_ was determined > 10 µM for A3, and > 30 µM for hNav1.5 and hGABAAα1β2γ2-gated Cl− channel, and there was no effect up to 30 µM on NK2. All tested targets and results are shown in Supplementary Table S1.

**Figure 2:**
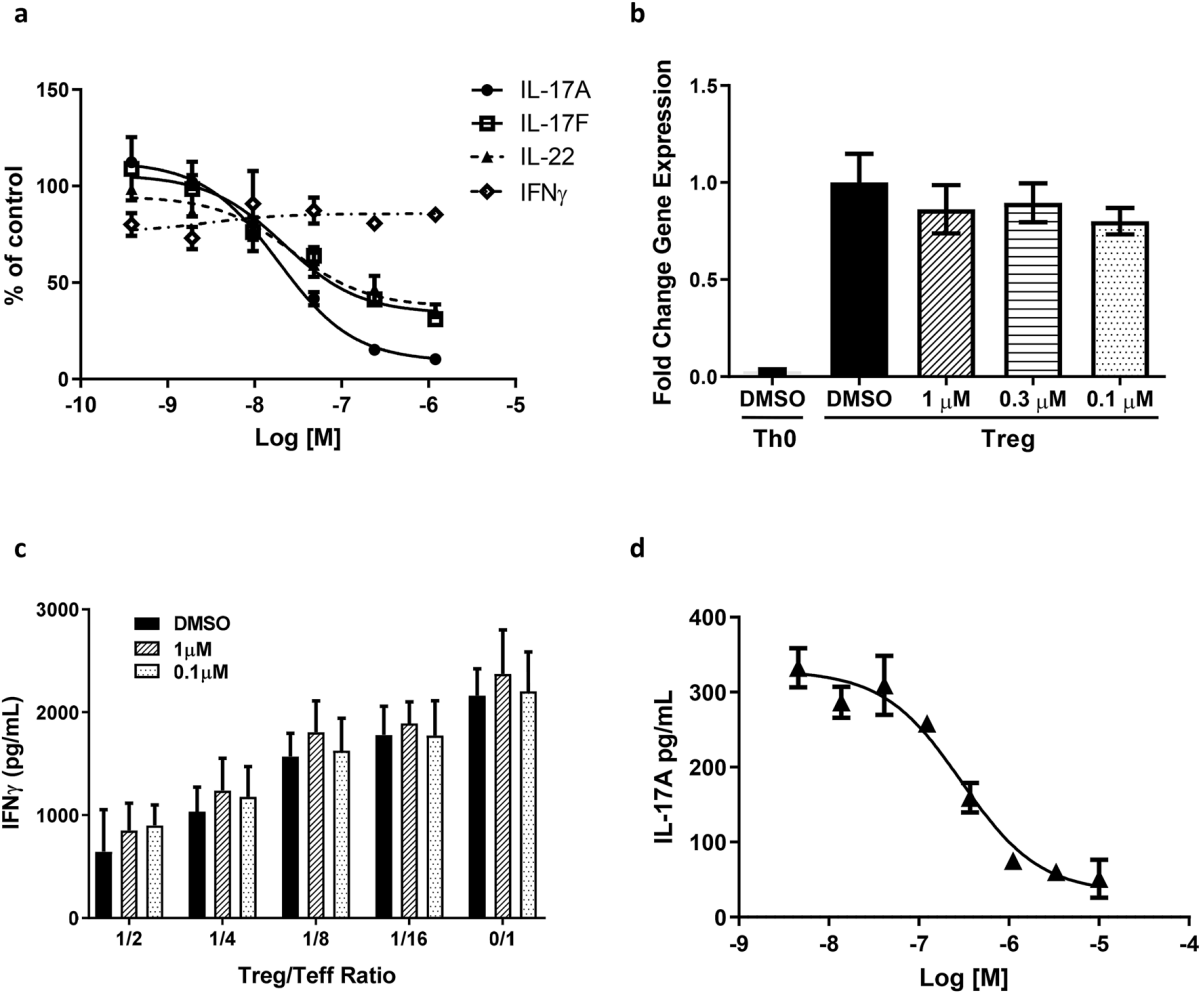
Effects of JNJ-61803534 on human immune cells. **(a)** Effect of JNJ-61803534 on IL-17A, IL-17F and IL-22 production in human CD4^+^ T cells under Th17 differentiation, and on IFNγ production in human CD4^+^ T cells under Th1 conditions, respectively. Data are averages of duplicates and presented as the percentage of vehicle control group. **(b)** Effect of JNJ-61803534 on FOXP3 gene expression after 6 days under Treg differentiation conditions. Data are presented as fold change in gene expression over vehicle control group (mean ± SD, n = 3). **(c)** Effect of JNJ-61803534 on Treg suppression of IFNγ production from effector T cells. Data are presented as mean ± SD, n = 3. **(d)** Dose-dependent inhibition of IL-17A production in 1:1 diluted human whole blood by JNJ-61803534. Data are average of duplicates.

RORγt drives Th17 differentiation and IL-17A, IL-17F and IL-22 production. To further evaluate its functional activity and selectivity, JNJ-61803534 was tested in total CD4^+^ T cells that were isolated from human blood, cultured under Th17- or Th1-polarizing conditions. On day 4, supernatants were analyzed for IL-17A, IL-17F, IL-22 and IFNγ production. JNJ-61803534 dose-dependently suppressed production of IL-17A, IL-17F and IL-22 with IC_50_ (95% confidence intervals) values of 19 (14–26) nM, 22 (8–62) nM and 27 (13–55) nM, respectively, and with the greatest level of inhibition of IL-17A under Th17-polarizing conditions, but showed no inhibition of IFNγ productions under Th1 conditions (Fig. 2a). The effect of JNJ-61803534 on Tregs was also evaluated. In vitro differentiation to Tregs was demonstrated by enhanced gene expression of FOXP3 in CD4^+^ T cells after being cultured for 6 days under Treg polarizing conditions, comparing to under neutral activation. The FOXP3 expression levels were similar in JNJ-61803534-treated and DMSO-treated cells (Fig. 2b), suggesting that JNJ-61803534 did not impact in vitro Treg differentiation. In addition, we tested the effect of JNJ-61803534 on natural Treg (nTreg) function. nTreg CD4^+^CD25^+^ cells, isolated from human peripheral blood, were co-cultured with monocyte-derived dendritic cells and CD4^+^CD25^-^ T effector cells (Teff) for 3 days, to test for suppression of Teff proliferation. nTregs displayed similar suppression of Teff cell proliferation (Supplementary Figure S1) and IFNγ production (Fig. 2c) in the presence of JNJ-61803534 at 1 µM and 0.1 µM compared with DMSO control, suggesting that JNJ-61803534 did not impair human nTreg suppressive function.

The activity of JNJ-61803534 was also tested in whole blood of human, mouse, and rat under conditions that favor Th17 activation and differentiation. Similar dose-dependent inhibition of IL-17A production was observed across species with average IC_50_ of 230 ± 110 nM, 172 ± 50 nM and 120 ± 10 nM in human, mouse, and rat whole blood, respectively. A representative dose response curve from a human blood assay is shown in Fig. 2d.

### In vivo pharmacology

#### Pharmacokinetics and pharmacodynamics

**Figure 3:**
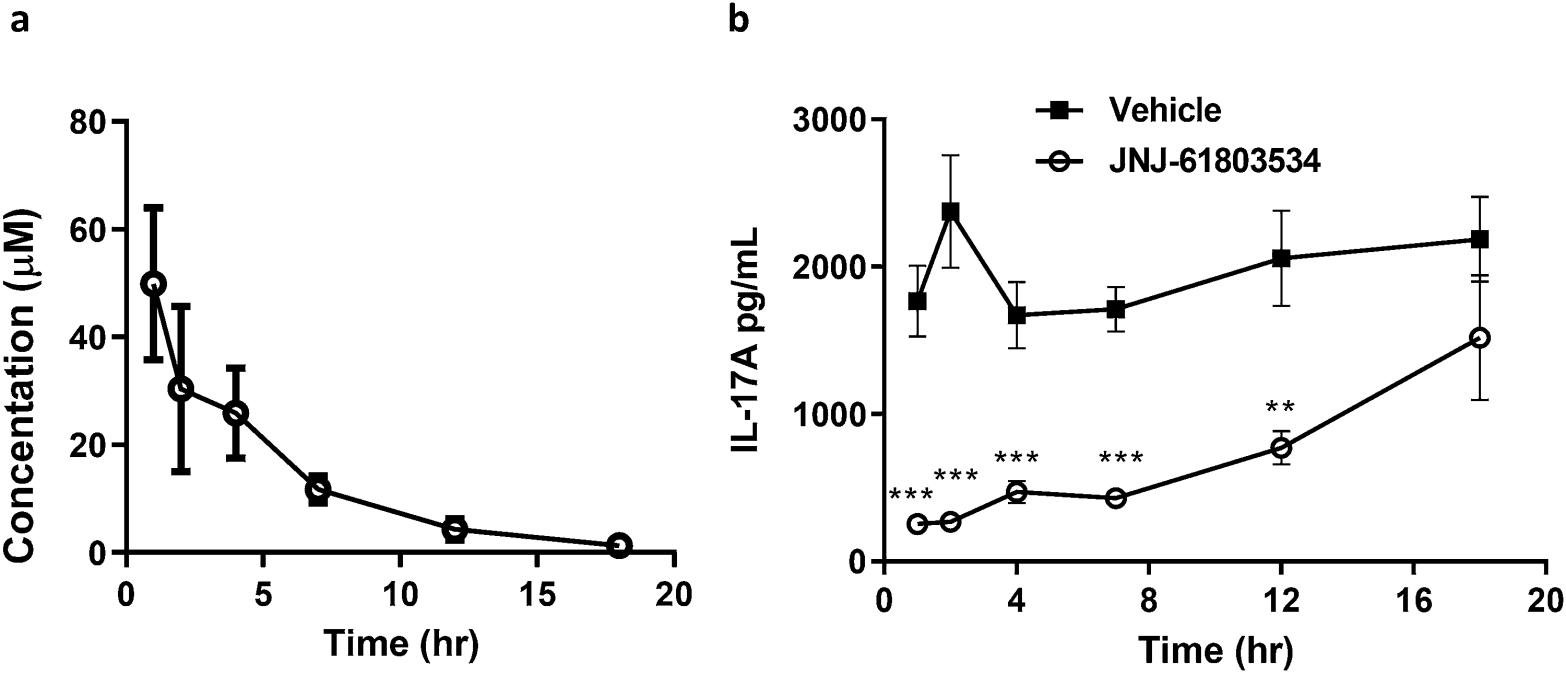
Effect of JNJ-61803534 in a mouse pharmacokinetics (PK)/pharmacodynamic (PD) model. **(a)** Time course of JNJ-61803534 concentration in plasma, and **(b)** Time course of inhibition of ex vivo stimulated IL-17A production by JNJ-61803534, at 100 mg/kg dosed orally. IL-17A values are presented as mean ± SEM of each time point of vehicle and treated group (n = 10). Unpaired t-test: *p < 0.05, **p < 0.01, ***p < 0.001, and ****p < 0.0001.

Mice were dosed orally with 100 mg/kg JNJ-61803534 and blood was collected at 1 h, 2 h, 4 h, 7 h, 12 h and 18 h for IL-17A analysis. JNJ-61803534 showed time-dependent exposures in plasma (Fig. 3a) and inhibited ex vivo stimulated IL-17A production in the blood (five-fold diluted in the assay) by 86 ± 6.7%, 89 ± 7.1%, 72 ± 14%, 75 ± 7.5%, and 62 ± 17% at 1 h, 2 h, 4 h, 7 h and 12 h with statistical significance, and 30 ± 60% at 18 h without statistical significance, when compared to corresponding vehicle treated groups (Fig. 3b). Compound concentrations are also listed as undiluted (measured) and diluted (extrapolated) in Supplementary Table S2. Compound concentration for 50% inhibition of ex vivo IL-17A was between 0.24 and 0.86 µM.

#### Mouse collagen-induced arthritis model

**Figure 4:**
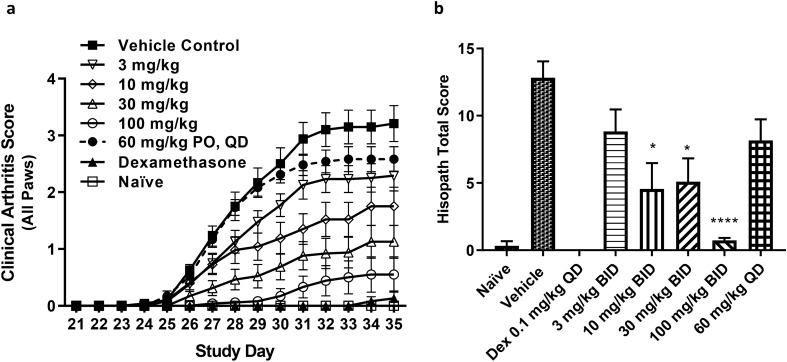
Effect of JNJ-61803534 in a mouse CIA model. **(a)** Time course of clinical arthritic score. **(b)** Histopathology score of the paws at the end of study. JNJ-61803534 was dosed orally at 3, 10, 30, 100 mg/kg/day, BID, or 60 mg/kg QD from day 21–35. Data represent mean ± SEM (n = 9–12 per group except naïve group n = 4, and Dex group = 6). *p < 0.05, ****p < 0.0001 vs Vehicle (Kruskal–Wallis, Dunn’s Multiple Comparison post-test).

To investigate the role of RORγt in innate and adaptive immune responses in vivo, we examined the effects of JNJ-61803534 in a mouse collagen-induced arthritis (CIA) model where mice were sensitized then challenged with collagen/CFA on day 1 and day 21, respectively. JNJ-61803534 was orally administered at 3, 10, 30, 100 mg/kg BID or 60 mg/kg QD, from day 21 (right before challenging) through day 35 (dosing only once on day 35). Compared to the vehicle group, the treatment with JNJ-61803534 showed significant dose-dependent reduction in disease scores from day 26 to day 35 (Fig. 4a), and hind paw histopathology scores including inflammation, cartilage damage, bone destruction, and remodeling of decalcified bone section on day 35 (Fig. 4b). Clinical arthritis scores, expressed as area under the curve (AUC), were significantly reduced in mice treated twice daily with 3 mg/kg (30%), 10 mg/kg (44%), 30 mg/kg (66%) and 100 mg/kg (88%), but only slightly reduced in mice treated with 60 mg/kg once daily (13%, p > 0.05), compared to vehicle controls (Supplementary Figure S2a). Vehicle control mice achieved 100% disease incidence by day 27 which remained consistent through day 35. Disease incidence at day 35 was 100% for 3 mg/kg, 89% for 10 mg/kg, 80% for 30 mg/kg, 45% for 100 mg/kg, and 100% for 60 mg/kg QD (Supplementary Figure S2b). JNJ-61803534 plasma concentrations were analyzed at two time points, 1 h and 12 h post last dose on study day 35. PK/PD modeling was used to simulate the plasma concentrations of compound for each group and exposure vs efficacy analysis result is shown in Supplementary Table S3.

### Imiquimod-induced dermal psoriatic-like inflammation in mice

**Figure 5:**
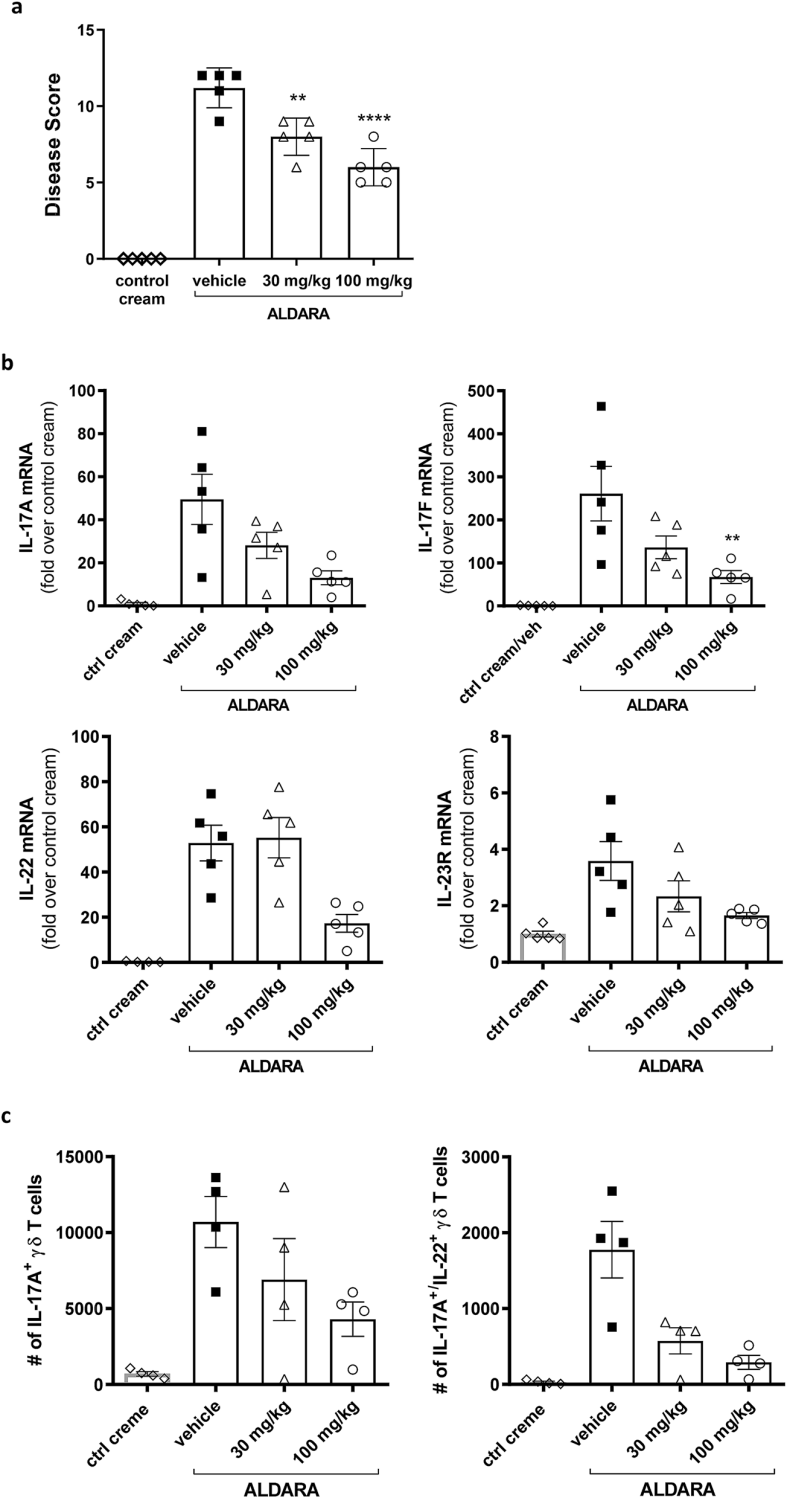
Effect of JNJ-61803534 in IMQ-induced skin inflammation in mice. **(a)** Cumulative skin scores on day 7 of the mouse IMQ model. Disease scores (thickness, redness, scaling) of back skin were measured prior to each morning dose and presented as mean ± SEM of 5 individual animal. **(b)** Gene expression (IL-17A, IL-17F and IL-22) in the ears of the mice. Data are presented as mean ± SEM of fold change over control cream group for each individual animal (n = 5). The relative expression level was calculated based on the formula: 2^ (β2M CT-Target Gene CT) * 10,000. **(c)** Number of IL-17A^+^ and IL-17A^+^/IL-22^+^ γδ T cells in the ears. Statistical analyses were performed with one-way ANOVA, *p < 0.05, **p < 0.01, ***p < 0.001, and ****p < 0.0001.

Imiquimod (IMQ) application to skin causes psoriasis-like skin inflammation in mice^46^ and humans^47^ through the Toll-like receptor-mediated innate immune response. IMQ induced skin inflammation in mice was used as a preclinical model of psoriatic inflammation. JNJ-61803534 was administered orally at 30 and 100 mg/kg to mice, and the inflammatory response to IMQ challenge was examined. JNJ-61803534 significantly reduced the disease scores (thickness, redness, scaling) of back skin in a dose-dependent manner (Fig. 5a). At the RNA level, JNJ-61803534 significantly inhibited IMQ-induced expression of IL-17A, IL-17F, and IL-22 genes at 100 mg/kg and also showed a trend towards inhibition of IL-17A and IL-17F expression at 30 mg/kg and IL-23R at 30 and 100 mg/kg, respectively (Fig. 5b). IL-10 expression was increased upon IMQ challenge and was not inhibited with JNJ-61803534 treatment, instead, we observed a trend towards further increases in IL-10 in a dose-dependent manner. In contrast, IFNγ expression was low and minimally impacted by IMQ or compound treatment (Supplementary Figure S3a). Infiltrated cells in the ear skin and draining lymph nodes were analyzed by flow cytometry, and showed that both IL-17A- and IL-17A/IL-22-producing γδ T cell populations were significantly increased by IMQ challenge and reduced by JNJ-61803534 in a dose-dependent manner (Fig. 5c and Supplementary Figure S3b-d). IMQ-induced neutrophils and inflammatory monocytes infiltrations in the ear were also significantly reduced at 100 mg/kg (Supplementary Figure S3e).

### Toxicology

JNJ-61803534 toxicology profile was evaluated in 1-month GLP studies in rats and dogs with an additional 1-month recovery period. The compound was orally administered as an aqueous suspension with citrate phosphate buffer (pH = 3.4) and 0.15% Xiameter. Rats generally tolerated the compound well and the highest dose tested of 400 mg/kg/day with C_max_ 29,600/24,200 ng/mL and AUC_0-24 h_ 310,000/376,000 ng.hr/mL for male and female, respectively, was considered the no-observed-adverse-effect level (NOAEL). Dogs were orally dosed at 3, 10, and 30 mg/kg/day. The 10 mg/kg/day dose was clinically well tolerated during the first 3 weeks of treatment then started to lose weight and reduce food consumption in the fourth week of dosing and a trend for increased alkaline phosphatase (ALP) at the end of study. 30 mg/kg/day exceeded the maximum tolerated dose, causing intestinal mucosal hemorrhages and slight hepatocellular lipid vacuolation in some animals. The 3 mg/kg dose with C_max_ 7250/7500 mg/mL and AUC_0-24 h_ 118,000/142,000 ng.hr/mL for male and female, respectively, was defined as the NOAEL. In both rat and dog studies, dose-dependent lymphocyte apoptosis in thymus was observed and was recovered after 1-month recovery period. The potential genetic toxicity of JNJ-61803534 was assessed in vitro in bacterial reverse mutation assay (AMES test) and mammalian cell (TK6) micronucleus test, and in vivo rat micronucleus test on bone marrow and was found to be all negative. In cardiovascular, respiratory, and central nervous system behavioral studies, JNJ-61803534 was well tolerated and had no indications of off-target effects that limit further use. In conclusion, JNJ-61803534 can be safely administered by the oral route. The signs of toxicity are monitorable and occur at exposures that exceed those predicted for efficacy in humans (50–60% inhibition of ex vivo stimulated IL-17A production at 24 h post-dose was predicted to require compound exposure at C_max_ ~ 1000 ng/mL and AUC ~ 65,000 ng.hr/mL), therefore, the first in human (FIH) study was conducted. Human dose equivalent to dog NOAEL (3 mg/kg/day) was calculated as 100 mg/day, and human starting dose was determined as 10 mg/day after applying ten-fold safety factor.

### Human clinical studies

The preclinical toxicology profile combined with the pharmacology data, indicating the safety and potential benefit in the treatment of a variety of inflammatory diseases, prompted the initiation of a clinical study to evaluate the safety, pharmacokinetics, and pharmacodynamics of JNJ-61803534 in healthy subjects. 48 participants were enrolled in a single ascending dose study, of which 47 completed the study and 1 participant in the placebo group withdrew consent. The baseline demographic and characteristics for each cohort are shown in Supplementary Table S4.

### Safety summary

There were no treatment-emergent adverse events (TEAEs) leading to death, treatment-emergent severe adverse events, severe TEAEs or TEAEs leading to discontinuation of the study agent during the study (Table 1). The TEAEs which were reasonably related to the study agent were reported for 1 participant each from the placebo group (presyncope and syncope) and the combined JNJ-61803534 group (diarrhea). There were transient changes in the hematology that were not considered TEAEs by the investigator or the sponsor medical monitor. Fourteen participants from the combined JNJ-61803534 group and 3 participants from the placebo group were found to have Grade 2 or higher abnormal cholesterol values. There was one participant from the placebo group, 2 participants from the JNJ-61803534 100 mg fasted group, and one from the JNJ-61803534 200 mg fasted group with clinically significant high cholesterol values. All these participants had high cholesterol values at screening, baseline, or both and the increases above these pre-dose values were transient and minimal. These changes were judged not clinically significant by the sponsor medical monitor. There were no clinically significant changes reported for other chemistry values. There were no clinically significant changes for urinalysis, vital signs, physical examination, or ECG. Overall, JNJ-61803534 was safe and well-tolerated as single doses up to and including 200 mg.

**Table 1:** Treatment-emergent adverse events with an incidence of > 1 in treatment groups after a single oral dose of JNJ-61803534, follow-up 42–57 days.

	Placebo n = 12	10 mg n = 6	30 mg n = 6	100 mg (fasted) n = 9	100 mg (fed) n = 9	200 mg n = 6
Diarrhea	0	0	0	2	2	0
Aphthous ulcer	0	0	0	0	1	1
Medical device site reaction	1	0	1	2	2	1
Back pain	0	1	0	1	1	1
Musculoskeletal discomfort	0	0	0	0	0	2
Viral upper respiratory tract infection	1	0	0	2	2	0
Oral herpes	0	0	1	0	1	0
Headache	2	0	1	4	0	0
Hypercholesterolaemia	1	0	0	2	0	1
Nasal congestion	0	0	0	2	0	0

### Pharmacokinetics

**Figure 6:**
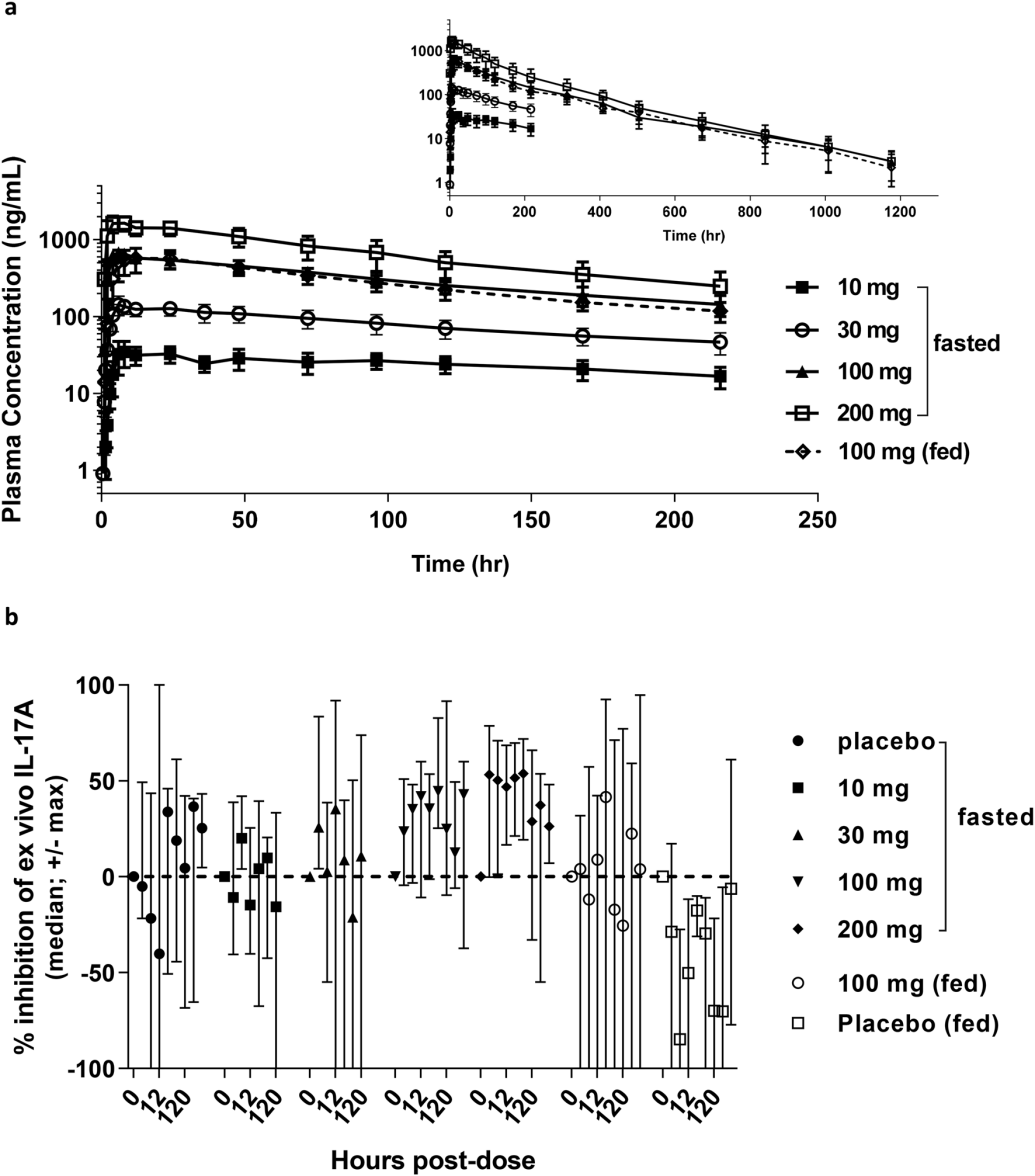
Pharmacokinetics and pharmacodynamics of JNJ-61803534 in healthy subject SAD phase I study. **(a)** Plasma concentrations of compound versus time. Data presented as mean ± SD. **(b)** Percentage of ex vivo IL-17A inhibition versus time, after single dose administration or placebo under fasted or fed conditions. Data are presented as median ± maximum/minimum. For both analyses, n = 6 for 10, 30 and 200 mg groups, n = 9 for 100 mg fed and fasted groups, n = 9 for placebo fasted group, and n = 3 for placebo fed groups.

The single dose JNJ-61803534 pharmacokinetic (PK) characteristics were evaluated at doses ranging from 10 to 200 mg with a 10 mg or 100 mg oral tablet under fasting (cohort 1–4) or fed (cohort 5) conditions (Fig. 6a and Supplementary Table S5). Mean JNJ-61803534 plasma concentrations increased with dose. After reaching maximum levels, JNJ-61803534 concentrations declined slowly, and also roughly in parallel across the dose groups. JNJ-61803534 C_max_ increased more than proportionally to the dose, across the evaluated 10 to 200 mg dose range. Based on mean values, dose normalized overall exposure (AUC_216h_, AUC_∞_) also appeared to increase proportionally with dose. Between subject variability (%CV) in C_max_ and AUC was modest, with values across the 5 cohorts ranging between 16.3 and 35.9% for C_max_, between 20.8 and 30.2% for AUC_216h_ and between 17.6 and 33.4% for AUC_∞_.

Median T_max_ was similar across dose groups, with a value of 6 h post-dose for the 30, 100, and 200 mg doses. For the 10 mg dose, median T_max_ was somewhat later, at 10 h post-dose. Mean T_1/2_ values were consistent across the dose groups, with mean values of approximately 1 week (163.8 to 169.9 h).

For the 100 mg dose, mean JNJ-61803534 plasma concentrations were overall similar after intake under fed or fasted conditions (Supplementary Table S5b). Absorption appeared to be slightly faster under fasted conditions, resulting in the mean maximum concentrations to be higher and reached earlier (6 h) compared to fed conditions (12 h).

### Pharmacodynamics

Blood samples were collected at various time points and dose-related pharmacodynamic (PD) effects of JNJ-61803534 were evaluated in an assay of ex vivo stimulated IL-17A production. The sampling was performed up to 120 h post-dose for cohort 1 (10 mg) and cohort 2 (30 mg), and extended to 504 h for cohort 3, 4 and 5.

Significant variability in IL-17A concentrations between subjects and between sampling time points within individual subjects was observed. IL-17A inhibition for the 10 and 30 mg doses did not appear to show a separation from placebo, while the 100 and 200 mg dose groups (cohort 3 and 4) showed maximum inhibition of 45% and 54% respectively, as compared to placebo (Fig. 6b). The 200 mg group (cohort 4) showed the most consistent inhibition across subjects from 2 to 48 h post-dose, while the 100 mg group (cohort 3) had more variable inhibition levels between time points. For the 100 mg dose, a higher inter-subject variability was observed in cohort 5 (fed intake) than cohort 3 (fasted intake). The scatter plot of IL-17A inhibition versus JNJ-61803534 plasma concentrations (Supplementary Figure S4) showed the concentrations corresponding to the 200 mg dose group achieved the higher end of the inhibition range.

400 mg and 600 mg SAD dose cohorts were originally planned, however, after the completion of the 200 mg SAD cohort 4, PK modeling based on available PK suggested that 400 mg would exceed the lowest-observed-adverse-effect level limits. Therefore, SAD was dosed only up to 200 mg. In addition, although a multiple ascending dose (MAD) study was planned after SAD, further clinical development was terminated, based on findings in a rabbit embryo-fetal study where fetal development was impacted by the treatment with JNJ-61803534.

## Discussion

RORγt plays a critical role in driving Th17 cell differentiation and expansion, as well as IL-17 production in innate and adaptive immune cells, making it an attractive therapeutic target for modulating diseases associated with the IL-23/IL-17 pathway. Here we describe the preclinical and clinical characterization of a novel selective RORγt inverse agonist.

JNJ-61803534 is highly selective against other nuclear receptors (NR) including RORα or RORβ, two closely related NR members, as well as a broad range of additional targets screened in selectivity panels. This compound inhibited the production of Th17 cytokines such as IL-17A, IL-17F and IL-22 but had no effect on Th1 cytokine, IFNγ, or Treg transcription factor FOXP3 expression, demonstrating its on-target functional activity and specificity. This is consistent with our previous finding with a structurally distinct molecule where no impact was observed on Th1 and Treg populations that were analyzed in addition to protein or mRNA measurements^22^. In addition, JNJ-61803534 did not affect nTreg suppressive activity on Teff cells, as evaluated by Teff proliferation and IFNγ production. It has been reported that Tregs can convert into IL-17-producing FOXP3^+^RORγt^+^ CD4^+^ T cells under certain microenvironments^48^. This IL-17-producing FOXP3^+^ Treg population, considered as an intermediate differentiation stage that can transform to Th17 cells, has been demonstrated both in mouse and human^48^. While the cell populations in the current study were not examined by co-staining FOXP3 and RORγt, treatment of activated CCR6^+^ T cells from human peripheral blood with RORγt inhibitor JNJ-54271074 in a previous study, slightly increased IL-10 production while greatly reducing IL-17A production^22^, suggesting skewing towards Tregs by inhibition of RORγt. Similarly, in the IMQ mouse model in the current study, JNJ-61803534 showed no inhibition and even showed a trend towards increasing IL-10 expression, while inhibiting RORγt-regulated genes in the inflamed tissue. Based on findings for these two structurally distinct RORγt inhibitors, we speculate that RORγt inhibition may block the conversion of Tregs to the intermediate IL-17-producing Treg and to Th17, and therefore may maintain or even increase IL-10-producing FOXP3^+^ Tregs.

JNJ-61803534 was tested for its inhibitory activity on stimulated IL-17A production in two-fold diluted human blood. As expected, the potency was right shifted about ten-fold in whole blood compared to isolated CD4^+^ cells due to significant plasma protein binding (98.9%). A similar assay in five-fold diluted whole blood from mouse and rat showed IC_50_’s similar to human whole blood values, demonstrating cross-species activity. A mouse PK/PD model was used to evaluate in vivo target engagement of JNJ-61803534 and its exposure-activity relationship. Dose- and time- dependent compound plasma exposures as well as exposure-dependent inhibition of ex vivo IL-17A production were observed after oral dosing of the compound. The EC_50_ generated from this model was similar to in vitro MWB IC_50_ after correcting for the dilution factor, demonstrating the in vitro*/*in vivo correlation of the potency and the in vivo target engagement by JNJ-61803534.

CIA is an experimental disease model characterized by a T cell-dependent autoimmune joint inflammation. Th17 cells have been identified as a key pathogenic subset in this model by producing inflammatory cytokines, especially IL-17A, that are important for priming collagen-specific T cells and for collagen-specific immunoglobulin (IgG)2a production^49^. In our CIA studies, oral dosing of JNJ-61803534 significantly attenuated inflammation, and achieved maximum inhibition of 88% for final arthritic score, and 94% inhibition of total histopathology score, at a dose of 100 mg/kg, BID. The incidence and onset of the disease were also reduced and delayed in a dose-dependent manner. While mice dosed with 60 mg/kg QD received the same total daily dose of JNJ-61803534 as the 30 mg/kg BID group, no significant reduction of disease score was observed in the 60 mg/kg treated group, suggesting the efficacy was not driven by maximum or total exposure of compound. Exposure-efficacy analyses showed that trough concentration (C_trough_) was the driver and that maintaining C_trough_ above the in vitro IC_50_ would be necessary for significant efficacy. The CIA study results demonstrated that JNJ-61803534 can suppress the autoimmune associated joint inflammation in a chronic model via oral dosing, and are consistent with published data with other structurally distinct RORγt inhibitors^22,50,51^. Biologics targeting IL-17A or IL-17RA have shown efficacy in chronic arthritic diseases such as psoriatic arthritis^52–55^ and ankylosing spondylitis^56,57^. Targeting the IL-12/23 pathway is also beneficial in psoriatic arthritis^58–61^. It has been reported that RORγt^+^ iNKT and γδ-hi T cells are enriched within inflamed joints of spondyloarthritides patients and functions of these cells can be blocked with ex vivo treatment of an RORγt inhibitor^62^. Thus preclinical data with RORγt inhibitors and clinical precedents for therapeutics in the same pathway suggest that RORγt modulation may have potential in treating psoriatic arthritis and/or ankylosing spondylitis.

The role of the IL-23/IL-17 pathway in the pathogenesis of plaque psoriasis is clearly validated by the clinical success of antibodies neutralizing IL-23p19, IL-17A or the IL-17A receptor. Clinical proof-of-concept has been demonstrated in a small Phase 2a trial where 4 weeks of treatment with an RORγt inhibitor VTP-43742 showed efficacy in patients with moderate to severe plaque psoriasis, accompanied by the reduction of IL-17A and IL-17F up to 75% in patient serum^63^. IMQ has been reported to induce epidermal expression of IL-23, IL-17A, and IL-17F, as well as an increase in splenic Th17 cells^46^, and mice deficient for IL-23 or the IL-17 receptor are resistant to IMQ-induced psoriasis-like skin inflammation, demonstrating a pivotal role of the IL-23/IL-17 axis in the animal model. In our study, treatment with JNJ-61803534 inhibited IMQ-induced skin inflammation as evidenced by a reduction in skin disease scores and infiltration of neutrophils and monocytes into the local tissue, as well as exhibited dose-dependent inhibition of skin expression of RORγt target genes. Application of IMQ for 6-days also caused the infiltration of CD3^+^ T cells to the skin. The majority of CD3^+^ T cells were IL-17A^+^ and IL-17A^+^/IL-22^+^ γδ T cells that were also increased in lymph nodes. These IL-17A-producing γδ T cells have been described as γδ T17 cells, and can expand in lymph nodes and traffic to the skin where they persist as memory-like cells capable of rapid activation upon challenge^64^. JNJ-61803534 treatment inhibited imiquimod-induced RORγt-dependent IL-17A- and IL-17A/IL-22- producing γδ T-cells in the ears and lymph nodes. Altogether, the data demonstrated that JNJ-61803534 achieved in vivo target engagement and efficacy in a preclinical psoriasis model, providing support for its potential in treating human psoriasis. RORγ, the isoform of RORγt, was reported to be expressed in all major resident skin cell populations in human including epidermal keratinocytes and dermal fibroblasts, and expected to play a role in regulating local homeostasis^65^. In our IMQ skin model, we focused on evaluating the impact of RORγt inhibition on immune cells and we did not examine RORγ-expressing cells. Whether RORγ is expressed in skin cells in mouse as observed in human and what role of RORγ plays in skin inflammation would be interesting topics for further investigation.

Preclinical toxicity studies in rats and dogs with JNJ-61803534 have identified the maximum tolerated exposures and exposure related safety parameters. Increased apoptosis in the thymus was observed in compound dosed animals. This is consistent with the reported physiological role of RORγt as a critical molecule in controlling the survival of thymocytes^7,66^. JNJ-61803534-induced thymocyte apoptosis was reversible and was deemed non-adverse alterations. Chronic dosing up to 9 months in dogs (data not shown) caused increase in medium-sized thymocytes/mitoses, which was also reversible and considered as a regenerative response to increased apoptosis.

In the clinical SAD study, JNJ-61803534 was in general well-tolerated up to the 200 mg single dose and no safety issues were identified. Across the evaluated dose range, 10 to 200 mg, C_max_ increased more than dose proportionally. The reason for this lack of dose proportionality in C_max_ is not known. Overall exposure (AUC_∞_) showed no obvious deviation from dose proportionality. Mean T_1/2_ values were consistent across the dose groups, with values of approximately 1 week. Food showed only a minimal effect on JNJ-61803534 pharmacokinetics, based on the overall exposure between cohorts under fed and fasted conditions.

The PD biomarker IL-17A, produced by ex vivo stimulation, displayed relatively large fluctuations between sampling time points within each individual. Also, changes in IL-17A levels compared to pre-dose showed high inter-subject variability in both the active dose and placebo groups. The source of this variability is not well understood but likely reflects both inter-individual differences, as well as varying proportions of cells capable of producing IL-17A present in any given blood sample, and how responsive those cells are to induction of IL-17A production. This variability may have made it challenging to show any separation from placebo in the lower dose 10 and 30 mg cohorts. More consistent IL-17A inhibition was observed in the 100 and 200 mg cohorts, and the 200 mg cohort showed the most consistent inhibition from 2 to 120 h, demonstrating target engagement-driven downstream PD effect in humans after a single oral dose of JNJ-61803534. Due to early discontinuation of the study, the PD effects of repeat dosing could not be assessed, and impact on endogenous serum level of IL-17A in psoriasis patients could not be further explored.

In conclusion, JNJ-61803534 is a potent and selective RORγt inhibitor that has demonstrated robust pharmacological inhibition of the IL-23 and IL-17 pathways preclinically and exhibited evidence of a pharmacodynamic effect in humans. Despite the termination of this compound due to the rabbit embryofetal toxicity findings, the data from our study and others, including the Phase 2a result of VTP-43742, as well as clinical efficacy of multiple biologics in the IL-23/Th17 pathways, all suggest that targeting RORγt has potential to deliver safe and effective treatments for a variety of immune-mediated conditions.

## Materials and methods

### One-hybrid reporter assay

The assay was carried out by transiently co-transfecting HEK293T cells with pFR-Luc reporter and pRL-CMV reporter (Promega #E2261), pCMV-BD (Stratagene #211342) containing the GAL4 DNA-binding domain fused with full-length human RORγt (Genbank accession no. NP_001001523, aa 1–497), or RORα (Genbank accession no. NP_599022, aa 305–556) or pCMV-BD-RORβ (Genbank accession no. NP_008845, aa 201–459). The transfected cells were cultured in the presence or absence of JNJ-61803534 for 16–20 h, after which firefly luciferase signals were measured, as described previously^22^.

### Selectivity assays

Selectivity against 18 nuclear receptors was tested for JNJ-61803534 in 1-hybrid reporter assays using a similar protocol to that described earlier, or in a biochemical TR-FRET assay^22,41^. 28 GPCRs were evaluated through cAMP or calcium FLIPR assays^67^. A panel of 52 different receptors, ion channels and transporters was tested by Cerep, Inc. (Redmond, WA). Kinase selectivity was conducted by Eurofins Panlabs Inc (St Charles, MO).

### Human blood

Preclinical samples were provided by the Scripps Research Institute. Blood donors, both male and females, have given informed consent to participate in this study and the study protocol on human blood samples was submitted by Janssen R&D and approved by the Scripps Research Institute IRB (Institutional Review Board). All experiments were performed in accordance with the relevant guidelines and regulations.

### In vitro human Th17, Th1 and regulatory T cell differentiation

Total CD4^+^ T cells were isolated from peripheral blood mononuclear cells (PBMCs) of healthy donors using a CD4^+^ T cell Isolation Kit II (Miltenyl Biotec, Auburn, CA), following the manufacturer’s instructions. Isolated CD4^+^ T cells were cultured under Th17, Th1 or regulatory T cell (Treg) differentiation conditions in the presence or absence of JNJ-61803534 (see supplementary materials and methods).

### Human nTreg suppressive assay

Frozen purified human CD4^+^CD25^+^ natural Treg cells (nTreg), monocyte-derived dendritic cells (DC) and CD4^+^CD25^-^ T effector cells (Teff) (Allcells, LLC, Alameda, CA) were thawed and co-cultured in the presence or absence of JNJ-61803534. T cell proliferation and IFNγ production were measured to evaluate nTreg activity (see supplementary materials and methods).

### Mice and rats

Animals were handled following the protocol approved by Janssen R&D La Jolla Institutional Animal Care and Use Committee and in accordance with the relevant guidelines and regulations. All in vivo studies were carried out in compliance with the ARRIVE guidelines.

### In vitro whole blood assay

Heparinized human, rat or mouse whole blood was collected and diluted in RPMI 1640 medium (at 1:1 for human or 1:4 for rat and mouse), then stimulated with anti-CD3, anti-CD8 and IL-23 in the presence or absence of JNJ-61803534. Two days later, IL-17A levels were measured in culture supernatant samples (see supplementary materials and methods).

### Mouse pharmacokinetic and pharmacodynamic model

Various doses of JNJ-61803534 or vehicle were administered orally to female C57B6 mice (Charles River Laboratories, Hollister, CA), and at different time points post dosing, the mice were euthanized, and blood was collected into heparinized tubes (BD Microtainer) for measurement of compound levels by LC–MS analysis and ex vivo stimulated IL-17A using the whole blood assay described above.

### Mouse collagen-induced arthritis model

On Day 0, mice (female DBA/1Lacj, Jackson Laboratories; 8–10 weeks) were immunized at the base of the tail with an emulsion containing equal amounts of Chick Type II Collagen (Chondrex, Redmond, WA) and Complete-Freund’s adjuvant (CFA, Chondrex), 100 µg each per mouse, and administered a second immunization boost on day 21. On this day, animals were examined for clinical arthritis scores and randomized into treatment groups. JNJ-61803534 (3, 10, 30, or 100 mg/kg twice daily or 60 mg/kg once a day) or vehicle (20% HPCD) was given orally daily from day 21 to 34. Clinical arthritis scores were evaluated from day 21 to 34, and animals were euthanized under CO_2_ on day 35. Both hind paws from each animal were collected and fixed in 10% neutral buffered formalin for histopathology.

### Imiquimod-induced dermal inflammation model

The backs of BALBc male mice were depilated one day prior to the start of dosing (day 0). Vehicle or JNJ-61803534 at 30 mg/kg and 100 mg/kg (in 20% HPCD) was orally administered twice a day on days 1 through 6, and once in the morning on day 7 with a dosing volume of 10 mL/kg. On days 2 through 6, 2–3 h after oral dosing of JNJ-61803534, 50 mg Aldara cream (containing 5% IMQ, Meda AB, Sweden) was applied to the back and 5 mg Aldara was applied to both ears of each mouse. For control groups, an equal amount of Balea cream (DM Drogerie Markt, Wals, Austria) was applied to both the back and ears of the mice. There was no Aldara/ Balea treatment on the final day (day 7). Disease (skin thickness, degree of skin redness and scaling) was measured prior to each morning dose and scores of 0–4 were assigned, with a maximum possible score of 12. On day 7, after morning dose, animals were sacrificed, and blood was collected via cardiac puncture. Skin, ear, and lymph nodes were collected for gene expression or flow cytometry analysis (see supplementary materials and methods).

### Toxicology studies

JNJ-61803534 was evaluated in repeat-dose toxicity studies for 1 month duration in Sprague–Dawley rats and Beagle dogs. The type of study plan was reviewed and agreed by the Laboratory Animal Welfare Officer and the Animal Ethical Committee (officially known as Dierexperimentencommissie (DEC) Charles River Den Bosch), as required by the Dutch Act on Animal Experimentation (February 1997). Both rat and dog studies were performed in AALAC accredited Charles River Laboratories in the Netherlands. Studies in rats were conducted with 0, 25, 100, and 400 mg/kg per day (n = 10/sex/group) for 1 month. An additional 5 animals/sex in vehicle and high dose were allowed to continue for a 1-month treatment-free recovery period. Studies in dogs were conducted with 0, 3, 10, and 30 mg/kg per day (n = 4/sex/group) for 1 month. A treatment-free recovery period of 1 month (additional 2 dogs/sex/group) was included for both vehicle controls and high dose groups. JNJ-61803534, as amorphous spray-dried powder containing hydroxypropylmethylcellulose acetate succinate (HPMC-AS) LG polymer at a ratio of 3:7, was formulated as an aqueous suspension with citrate phosphate buffer (pH 3.4) and 0.15% Xiameter and administered daily by gavage. Rats and dogs were examined for mortality, clinical signs, ophthalmoscopic changes, body weight, food consumption, hematology, clinical chemistry, anatomic pathology, and toxicokinetics. In addition, dogs were examined for electrocardiographic changes. Rats and dogs were also assessed for reversal of any effects following a 1-month recovery period. These studies were conducted in compliance with good laboratory practice (GLP) regulations.

### Clinical single ascending dose study

Randomized, double-blind, placebo-controlled, single center study was designed by Janssen Immunology Clinical Development team and approved by Independent Ethics Committee/Institutional Review Board (Comité voor Medische Ethiek UZA, Wilrijkstraat 10, Edegem, 2650, Belgium). The study was conducted in Janssen’s Clinical Pharmacology Unit in Belgium from May 18th 2017 to April 5th 2018, according to Declaration of Helsinki principles and International Committee on Harmonisation Good Clinical Practice guidelines. The trial was to investigate the safety, tolerability, pharmacokinetics, and pharmacodynamics of JNJ-61803534, and registered as NCT03139500 on 04/05/2017 at ClinicalTrials.gov. Participants were healthy males and females of non-childbearing potential (postmenopausal or permanently sterile), aged 18 to 60 years inclusive, with a body mass index of 18 and 30 kg/m^2^ and body weight of not less than 50 kg. Informed consent was obtained from all participants. No formal sample size and power calculations were performed. The number of participants chosen for this study was considered adequate to provide a preliminary safety assessment and PK assessment. The randomization was based on a computer-generated treatment randomization schedule prepared and balanced by using randomly permuted blocks. Two blocks was utilized with the first block of size 2 and randomization ratio of 1:1 and the second block of size 6 and randomization ratio of 5:1 (JNJ-61803534: placebo). Participants and study site staff members including the Investigator were blinded to treatment allocation during entire study period. JNJ-61803534 was supplied as 10 mg and 100 mg tablets, which have no visual differences from placebo tablets. Cohorts 1 to 4 received single oral doses of 10, 30, 100 and 200 mg after an overnight fast, and cohort 5 were dosed 100 mg within 30 min of the start of a standard high fat meal. Participants were randomized to JNJ-61803534 (n = 6) or placebo (n = 2) for cohort 1, 2 and 4, and JNJ-61803534 (n = 9) or placebo (n = 3) for cohorts 3 and 5. The doses of JNJ-61803534 were escalated in a stepwise fashion if the safety, tolerability, and plasma pharmacokinetic profile were deemed acceptable. For all parts of the study, adverse events and concomitant medications were assessed and recorded from screening through follow-up (day 14–21 post-dose). The following safety measures were assessed at various time points during the study: medical history, physical examination, neurologic examination, electrocardiogram (12-lead ECG, or continuous telemetry), and vital signs (blood pressure, heart rate, respiratory rate, and temperature). Safety measures also included clinical laboratory tests: blood chemistry; hematology, coagulation, and serology tests; urinalysis; alcohol analysis; urine pregnancy test and serum pregnancy test (females); urine drug screen; 24-h urine for creatinine clearance, protein, and albumin excretion rate^68^. This was a Phase 1 first-in-human study with limited sample size, the data generated was descriptive in nature, and no formal statistical hypothesis testing was planned. Although there was no formal interim analysis, assessments of PK and PD samples by cohort and safety assessments were conducted and reviewed during the study to plan dose-escalation and future development.

### Pharmacokinetic evaluation

For all parts of the clinical study, venous blood samples were taken for the measurement of JNJ-61803534 plasma concentrations at pre-dose, 1, 2, 4, 6, 8, 12, 24, 48, 72, 96, 120, 168, 216, 312, 408, 504, 672, 840, 1008, and 1176 h post-dose. Plasma samples with K_2_EDTA anticoagulant were treated with acetonitrile for protein extraction then processed with liquid chromatography coupled to tandem mass spectrometry. Reversed-phase HPLC separation was used and tandem MS/MS detection was set in TIS positive mode. A stable labeled analyte was used as internal standard to determine concentrations of JNJ-61803534 and the lowest limit of quantification was 1 ng/ml. All the data reported in sample analysis and validation met the predefined acceptance criteria and fulfilled the requirements and recommendations in the current FDA guidance for bioanalytical method validations and analysis.

### Pharmacodynamic assay

Whole blood samples were collected on the morning of day 1 at pre-dose and at 1, 2, 6, 12, 24, 48, 96, and 120 h post-dose in Cohorts 1 and 2. For Cohort 3,4, and 5, samples were collected the morning of day 1 at pre-dose and at 2, 6, 12, 24, 48, 120, 312 and 504 h post-dose. Blood samples (1 ml) were drawn into TruCulture tubes (Myriad RBM, Austin, TX) which contained 2 ml of cocktail containing cell culture media and stimulants, then mixed and incubated at 37 ºC in a block thermostat for 48 h. The final concentrations of the stimulants in the TruCulture tube incubations were: IL-23 (50 ng/ml), and IL-1β (10 ng/ml), with anti-CD3 and anti-CD28 antibodies at standard concentration as per standard CD3/CD28 TruCulture tubes. Negative control tubes (no stimulants) were also collected at each timepoint. Supernatants were collected after the end of the incubation period and analyzed to determine concentrations of induced IL-17A using a validated immunoassay method (human IL-17A V-plex kit from Meso Scale Discovery).

## Supplementary Information

Supplementary Information.
