# Author Correction: Preclinical and clinical characterization of the RORγt inhibitor JNJ-61803534

**DOI:** 10.1038/s41598-022-09558-2

**Published:** 2022-04-06

**Authors:** Xiaohua Xue, Aimee De Leon-Tabaldo, Rosa Luna-Roman, Glenda Castro, Michael Albers, Freddy Schoetens, Samuel DePrimo, Damayanthi Devineni, Thomas Wilde, Steve Goldberg, Olaf Kinzel, Thomas Hoffmann, Anne M. Fourie, Robin L. Thurmond

**Affiliations:** 1 Janssen Research & Development, LLC, La Jolla, CA USA; 2 Janssen Research & Development, LLC, Spring House, PA USA; 3 Department of Research, Phenex Pharmaceuticals AG, Heidelberg, Germany

Correction to: *Scientific reports* 10.1038/s41598-021-90497-9, published online 26 May 2021

The original version of this Article contained errors.

Olaf Kinzel was omitted from the author list in the original version of this Article.

In addition, the Acknowledgments section now reads:

“We thank members of Immunology Discovery, Discovery Sciences and Clinical Development teams in Janssen Research and Development, LLC and members in Phenex Pharmaceuticals AG for contributions and support of this work. We especially thank Tinghua Cao, Marciano Sablad, Monika Banaszewska, and Thomas Schlueter for performing or coordinating some biological assays. We thank Tony Greway and William Barchuk for clinical study discussions.”

Furthermore, the Author Contributions section now reads:

“X.X. supervised biology studies and wrote the manuscript. A.D.-T., R.L.-R., and G.C. performed biology experiments. M.A. coordinated biology experiments and performed some data analysis. F.S. supervised 1-month toleration studies. S.D. supervised clinical PD assay. D.D. and T.W. modeled human PK/PD and doses. S.G. and O.K. supervised medicinal and synthetic chemistry work. T.H. coordinated in vivo study. R.L.T. led clinical study. X.X., F.S., S.D., D.D., T.W., S.G. and R.L.T. were involved in the clinical study design. F.S., S.D., D.D., T.W., S.G. A.M.F. and R.L.T. contributed to writing manuscript. All authors reviewed the manuscript.”

Finally, the original version of this Article contained an error in Figure 1 where the structure of the compound JNJ-61803534 was drawn with the incorrect methyl stereochemistry on the pyrrolidine ring in panel (a). The original Figure 1 and accompanying legend appear below.

**Figure 1:**
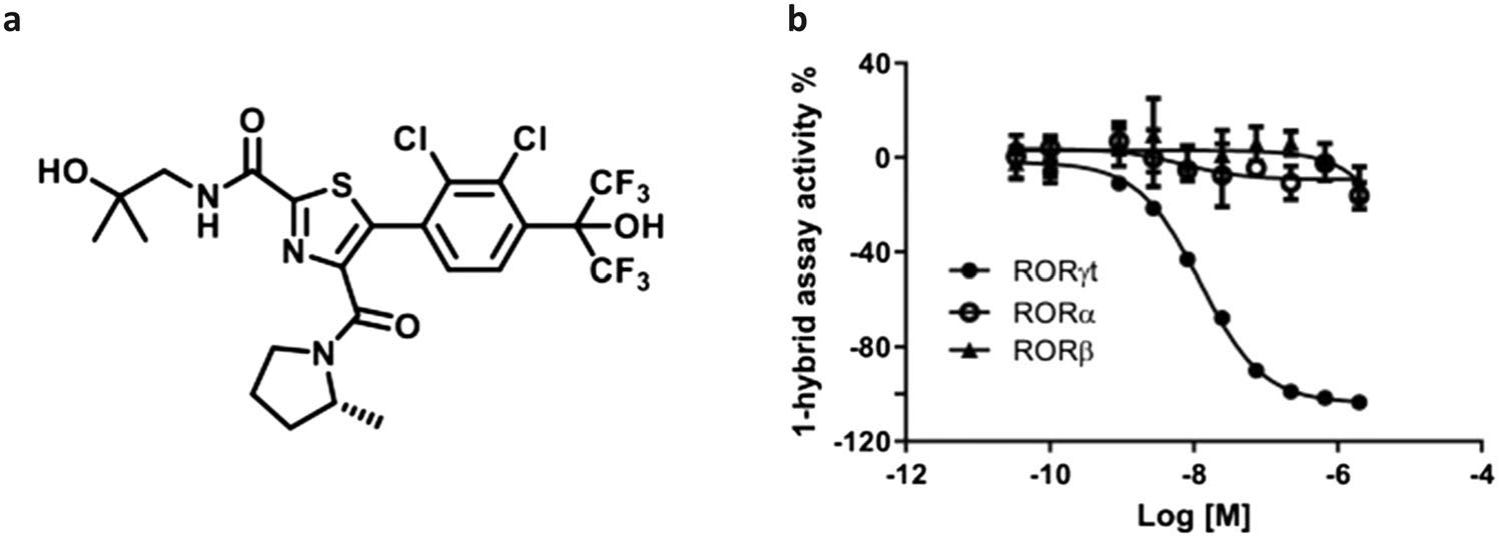
Structure and selectivity of JNJ-61803534 for inhibition of RORγt-driven transcription. (**a**) Structure of JNJ-61803534. (**b**) Activity of JNJ-61803534 in 1-hybrid reporter assays. HEK-293 T cells were transfected with vectors encoding RORγt, RORα or RORβ, respectively, fused with the GAL4 DNA binding domain. After incubation with compound overnight, luciferase signals were measured. JNJ-61803534 was tested at a starting concentration of 2 μM in three-fold serial dilutions in duplicate.

The original Article has been corrected.

