# Supplementary material for: Preclinical and clinical characterization of the RORγt inhibitor JNJ-61803534
Source: Sci Rep. 2021 May 26;11:11066. doi: 10.1038/s41598-021-90497-9 (PMC8155022; doi:10.1038/s41598-021-90497-9)
Supplement: Supplementary file 1 — Supplementary Information. [file 41598_2021_90497_MOESM1_ESM.pdf]

Preclinical and Clinical Characterization of the RORγt Inhibitor JNJ-61803534

Xiaohua Xue[1], Aimee De Leon-Tabaldo[1], Rosa Luna-Roman[1], Glenda Castro[2], Michael Albers[3], Freddy Schoetens[1], Samuel DePrimo[1], Damayanthi Devineni[2], Thomas Wilde[2], Steve Goldberg[1], Thomas Hoffmann[3], Anne M Fourie[1], Robin L Thurmond[1]**

[1]Janssen Research & Development, La Jolla, California, United States; [2]Janssen Research & Development, Spring House, Pennsylvania, United States; [3]Department of Research, Phenex Pharmaceuticals AG, Heidelberg, Germany

*Corresponding authors, email: xxue@its.jnj.com; RTHURMON@its.jnj.com

Supplement Materials and Methods

In vitro human Th17 and Th1 differentiation

Total CD4$^+$ T cells were isolated from the peripheral blood mononuclear cells (PBMCs) of healthy donors using a CD4$^+$ T cell Isolation Kit II (Miltenyl Biotec, Auburn, CA), following the manufacturer's instructions. Isolated T cells were resuspended in culture medium and added to a 96-well plate at 1×10^5 cells per well. Titrated JNJ-61803534 was added to each well, followed by addition of anti-CD3/CD28 beads (prepared using human T cell activation/expansion kit, Miltenyi Biotec) at final concentration of 3x10^6/mL, and cocktail for either Th17 differentiation (10 µg/mL anti-IL-4, 10 µg/mL anti-IFNγ, 10 ng/mL IL-1β, 10 ng/mL IL-23, 50 ng/mL IL-6, 3 ng/mL TGFβ and 20 U/mL IL-2), or for Th1 differentiation (10 µg/mL anti-IL-4, 10 ng/mL IL-12, and 20 U/mL IL-2). Cells were cultured at 37°C and 5% CO_2 for 3 days. Supernatants were assayed for accumulated cytokines using MSD multi-spot assay (Meso Scale Discovery, Rockville, MD) or R&D ELISA kit.

In vitro human Treg differentiation

Total CD4$^+$ T cells were isolated as described above, and seeded in a 96-well plate at 2×10^5 cells per well. Titrated JNJ-61803534 was added to each well, followed by addition of anti-CD3/CD28 beads at final concentration of 4x10^6/mL, and medium only for Th0 or cocktail for Treg differentiation at the final concentration of anti-IL-4 at 10 µg/mL, anti-IFNγ at 10 µg/mL, anti-IL-12 10 µg/mL, TGF-β1 10 ng/mL and IL-2 200 U/mL. Cells were cultured at 37°C and 5% CO_2 for 6 days, then cell pellets were collected and lysed and FOXP3 gene expression was measured using Quantigene 2.0 assay.

Human nTreg Suppressive Assay

Frozen purified human CD4+CD25+Treg cells (nTreg), monocyte-derived dendritic cells (DC) and CD4$^+$CD25$^-$ T effector cells (Teff) (Allcells, LLC, Alameda, CA) were thawed and co-cultured in RPMI+10%FBS containing 5 µg/mL anti-human CD3 antibody in a 96-well plate with the following density per well: 5x10^4 CD4$^+$CD25$^-$ Teff cells, 5x10^3 cells/mL of DC, and 2-fold serially diluted CD4$^+$CD25$^+$ nTreg cells to achieve the ratio of nTreg to Teff is 1/1, ½, ¼, 1/8, 1/16 and 0/1, in the presence of DMSO or titrated JNJ-61803534 at final DMSO concentration of 0.1% in 200 µL total volume. Cells were cultured at 37°C 5% CO_2 for 3 days, then 100 µL of supernatants were collected and measured for IFNγ production. 100 µL of fresh RPMI+10% FBS containing 1 µCi of ^{3}H-thymidine was added into each well

and was incubated overnight then cells were harvested ^3H thymidine incorporation was counted using Topcount NXT HT (PerkinElmer).

In vitro whole blood assay

For human blood assay, heparinized whole blood was collected from healthy volunteers and diluted 1:1 with RP RPMI1640 (R&D), then was added to a 96-well plate at 200 µl per well. DMSO or titrated JNJ-61803534 was added to the blood samples at 2 µL/well for a final DMSO concentration of 0.2% and incubated at 37°C for 1 hr. A 10 µL stimulation cocktail, was added to each well at the final concentrations: 1 µg/mL anti-CD28 (eBioscience), 50 ng/mL IL-23 (R&D systems), 10 ng/mL IL-1β (R&D systems). The blood was then transferred to an anti-CD3 pre-coated plate (1 µg/mL BD Biosciences) and incubated at 37°C for 2 days. For mouse blood assay, heparinized mouse blood was diluted 1:4 (5-fold dilution) in RPMI1640, stimulated with pre-coated anti-CD3 and soluble anti-CD8 (4 µg/mL each; R&D) and IL-23 (50 ng/mL; R&D) at 37°C for 42 hrs. Plasma was collected after incubation and human or mouse IL-17A levels were determined by ELISA, using commercial ELISA kits (R&D).

RNA extraction, quantitative RT-PCR

Frozen ear samples from IMQ model were homogenized in RNAzol®RT (Sigma-Aldrich) and were mixed with 200 µL RNase-free water and incubated for 10 min at room temperature. Samples were then centrifuged and ~500 µL supernatant was collected and mixed with 200 µL 75 % ethanol then transferred to one well of a RNeasy 96 well plate (Qiagen). Total RNA was prepared as described in the manual of the RNeasy 96 Kit.

The RNAs were reverse transcribed into cDNA using the Superscript II reverse transcriptase (Life Technologies), then real time PCR reactions (Taqman) were performed on an ABI Prism 7900HT Sequence Detection System using predesigned qPCR Assays (Integrated DNA Technologies, Inc.) containing validated probe/primer forward/primer reverse combinations for the individual genes: mIL-17A (TCCACCGCAATGAAGACCCTGATAG; AGACTACCTCAACCGTTCCA; GAGCTTCCCAGATCACAGAG); mIL-17F (ACCCGTGAAACAGCCATGGTCAA; TGGAGAAACCAGCATGAAGTG; TGGAGAAACCAGCATGAAGTG); mIL-22 (AGCCGTACATCGTCAACCGCA; AGCTTGAGGTGTCCAACTTC; GGTAGCACTGATCTTTAGCACTG). For calculation of relative changes in gene expression the ddCT algorithm (1) was applied and TATA-Box binding protein (TBP) was used for normalization.

Flow cytometry analysis

Single cell suspensions were prepared by incubating ear samples from IMQ model with 0.05 mg/mL DNase and 0.4 mg/mL Liberase (Roche) in RPMI 37°C for 1 h. Cells were stained with fluorescence-conjugated antibodies against surface markers for different cells types. Intracellular staining for IL-17A and IL-22 was performed on cells incubated with a leukocyte activation cocktail containing brefeldin A, PMA and ionomycin for 4 h and stained with anti-TCR antibodies. The stained cells were acquired with the flow cytometer Canto II (BD Biosciences), and the data was analyzed using the FlowJo version 9.7.5 (Treestar). The following mouse antibodies were used for staining: anti-CD45.2-APC-ef780, anti-$\gamma\delta$ TCR-FITC, anti-CD11b-Biotin, anti-F4/80-PE, purchased from eBioscience; anti-CD3-PerCp, anti-CD4-V450, anti-CD19-Biotin, anti-Gr1-APC, purchased from BD Biosciences; anti-IL-17A-APC (eBioscience) and anti-IL-22-PE (Biolegend).

Suppl Table S1

a

**Nuclear Receptor**	**JNJ-61803534<br>in agonist and antagonist mode**
TRα<br>RARα	> 1.5 µM<br>> 1.5 µM
PPARα	> 1.5 µM
PPARβ	> 1.5 µM
PPARγ	> 1.5 µM
LXRβ	> 1.5 µM
FXR	> 1.5 µM
VDR	> 1.5 µM
PXR	possibly partial modulator; IC_{50}: 340 nM; remaining PXR activation: 40-50%
CAR	> 1.5 µM
RXRα	> 1.5 µM
ERα	> 1.5 µM
ERβ	> 1.5 µM
ERRγ	> 10 µM
GR	> 1.5 µM
MR	> 1.5 µM
PR	> 1.5 µM
AR	> 1.5 µM

b

Target	% Inhibition			Target	% Inhibition	
	at 1 μM	at 10 μM			at 1 μM	at 10 μM
A1 (h)	-	-		Y1 (h)	-	-
A2A (h)	-	-		Y2 (h)	-	-
A3 (h)	-	45.2**		NT1 (h) (NTS1)	-	-
alpha 1 (non-selective)	-	-		delta 2 (h) (DOP)	-	-
alpha 2 (non-selective)	-	-		kappa (KOP)	-	-
beta 1 (h)	-	-		mu (h) (MOP) (agonist site)	-	12.2
AT1 (h)	-	-		ORL1 (h) (NOP)	-	-
BZD (central)	-	-		5-HT1A (h)	-	-
B2 (h)	-	-		5-HT1B	-	15.6
CCKA (h) (CCK1)	-	-		5-HT2A (h)	-	-
D1 (h)	-	-		5-HT2B	-	36.8
D2S (h)	-	-		5-HT3 (h)	-	-
ETA (h)	-	-		5-HT5A (h)	-	-
GABA (non-selective)	-	-		5-HT6 (h)	-	-
GAL2 (h)	-	-		5-HT7 (h)	-	-
CXCR2 (h) (IL-8B)	-	-		sst (non-selective)	-	-
CCR1 (h)	-	-		VIP1 (h) (VPAC1)	-	-
H1 (h)	-	-		V1a (h)	-	20.3
H2 (h)	-	16.6		Ca^{2+} channel	-	-
MC4 (h)	-	-		K+V channel	-	-
MT1 (h)	-	20.4		SK+Ca channel	-	-
M1 (h)	-	-		Na^{+} channel (site 2)	15	46
M2 (h)	-	-		Cl^{-} channel	10.5	86.4*
M3 (h)	-	-		NE-T transporter (h)	-	-
NK2 (h)	-	54.8*		DA-T transporter (h)	-	-
NK3 (h)	-	-		5-HT-T	-	-

c

Target	EC_{50} µM (agonist)	IC_{50} µM (antagonist)
5HT1A	>12.5	>12.5
5HT1B	>12.5	>12.5
5HT2A	>10	>10
5HT2B	>10	>10
5HT2C	>10	>10
5HT6	>12.5	>12.5
5HT7	>12.5	>12.5
A2A	>12.5	>12.5
A3	>12.5	>12.5
ADRA1A	>10	>10
ADRA2A	>12.5	>12.5
ADRA2B - AG	>12.5	>12.5
ADRA2C	>12.5	>12.5
ADRB1	>12.5	>12.5
ADRB2	>12.5	>12.5
AT1	>5	>5
CCKA	>10	>10
D1	>12.5	>12.5
D2L	>12.5	>12.5
delta	>12.5	>12.5
ETA	>10	>10
H1	>10	>10
KOP	>12.5	>12.5
M1	>10	>10
M2	>12.5	>12.5
M3	>10	>10
MOP	>12.5	>12.5
V1A	>10	>10

d

Kinase	Activity % @ 1 µM	Activity % @ 10 µM
ALK4(h)	104	108
Aurora-A(h)	97	102
Aurora-B(h)	124	145
Blk(h)	95	102
CaMKIIβ(h)	89	92
CaMKIδ(h)	81	100
CDK1/cyclinB(h)	96	108
CDK5/p35(h)	97	107
CHK1(h)	97	132
CHK2(h)	107	111
CK2(h)	109	102
CK2α2(h)	92	100
CLK2(h)	108	99
cSRC(h)	78	80
EGFR(h)	100	101
EphA2(h)	99	102
FGFR1(h)	103	125
Flt3(h)	84	103
GSK3α(h)	93	104
GSK3β(h)	111	105
IGF-1R(h)	95	106
IKKβ(h)	75	138
IR(h)	92	89
IRAK4(h)	113	112
JAK2(h)	109	105
JAK3(h)	109	114
MAPK1(h)	91	112
MAPK2(h)	124	127
NEK2(h)	102	108
PAK4(h)	104	103
PDGFRβ(h)	103	143
Pim-2(h)	102	107
PKA(h)	85	88
PKBα(h)	97	95
PKCα(h)	102	99
PKCβI(h)	97	87
Plk1(h)	111	104
Plk3(h)	120	114
Ret(h)	107	104
ROCK-I(h)	108	110
Rsk3(h)	107	104
SAPK3(h)	106	112
SAPK4(h)	100	112
TrkC(h)	92	88
ZAP-70(h)	137	119
ZIPK(h)	113	120

Legend for Suppl Table S1

Selectivity evaluation on JNJ-61803534. (a) nuclear receptors; (b) Cerep panel -receptors, transporters and ion channels. * >30 µM of IC_{50}, **>12.5 µM IC_{50}, in follow up functional assays; (c) GPCR panel; (d) Kinase panel

Suppl Figure S1

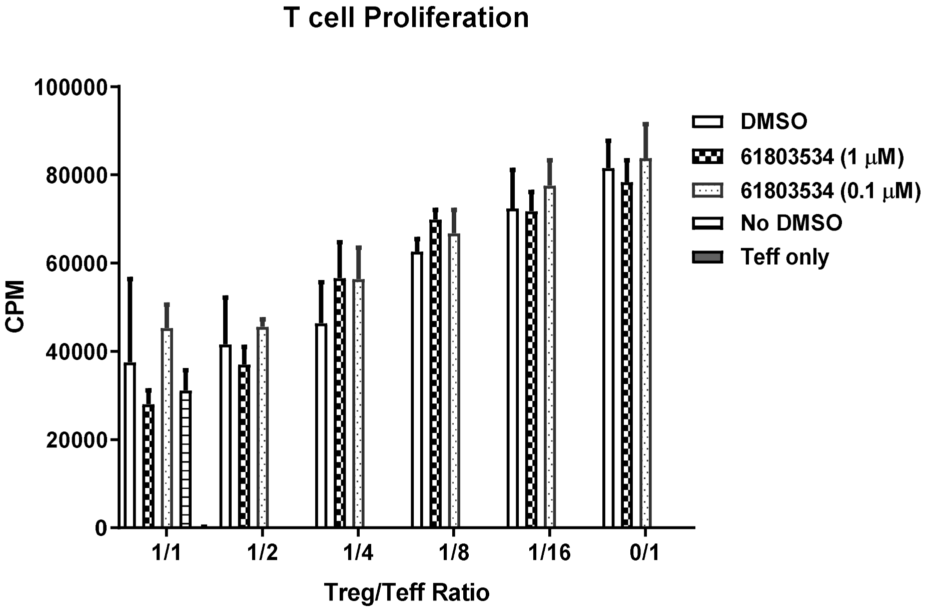

Supple Figure S1. Effect of JNJ-61803534 on Treg function for its suppression on the proliferation of effector T cells. Data are presented as mean ± SD, n=3.

Suppl Table S2

Time post-dose (hr)	In undiluted plasma		In 5-fold diluted plasma	% Inhibition of IL-17A (assayed in 5-fold diluted blood)
	ng/mL	μM	μM	
1	31022 ± 8809	49.8 ± 14.2	10.0 ± 2.8	86
2	18903 ± 9563	30.4 ± 15.4	6.1 ± 3.1	89
4	15469 ± 5234	24.9 ± 8.4	5.0 ± 1.7	72
7	7276 ± 1562	11.7 ± 2.5	2.3 ± 0.5	75
12	2666 ± 1237	4.3 ± 2.0	0.86 ± 0.4	62
18	759 ± 316	1.2 ± 0.5	0.24 ± 0.1	30

Suppl Table S2. Plasma exposure and percent inhibition of *ex vivo* IL-17A production at various time points after oral administration of 100 mg/kg JNJ-61803534. Exposure levels measured in undiluted plasma (in ng/mL and μM) and calculated for 5-fold dilution (in μM) are presented as mean ± SD (n=10)

Suppl Figure S2

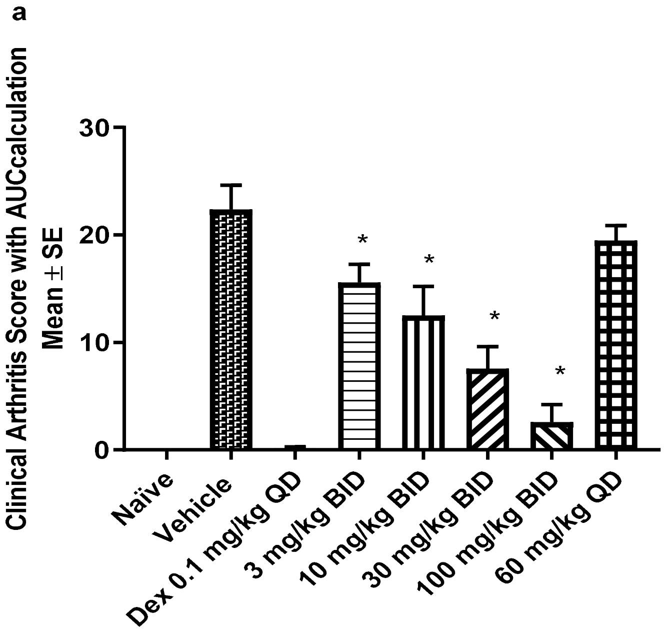

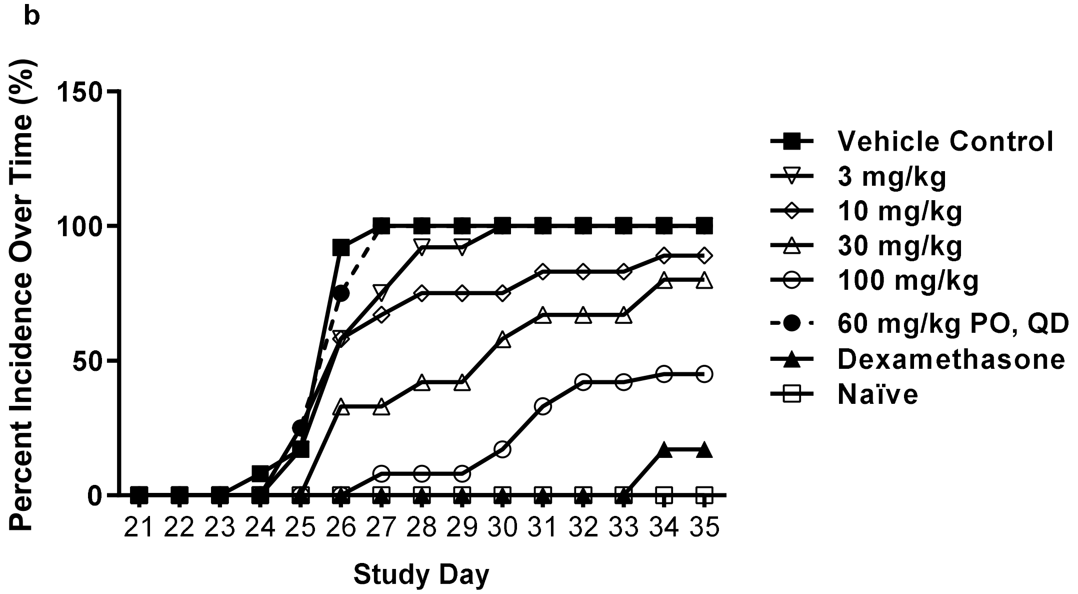

Suppl Figure S2. Effect of JNJ-61803534 (3, 10, 30, 100 mg/kg/day, PO, BID, and 60 mg/kg/day, PO, QD, day 21-35) of dosing in mouse CIA. (a) AUC of clinical arthritic score. Data represent mean + SE (n=9-12 per group except naïve group n=4, and Dex group =6). *p<0.05 vs Vehicle (Kruskal-Wallis, Mann Whitney U post-test. (b) Percent (%) incidence of disease over time. Percent incidence was calculated by dividing the number of animals with score >0 by the total number of living animals in the treatment group.

Suppl Table S3

Dose mg/kg	Dose Regimen	C_{trough} (µM)	$C_{average}$ (µM)	Efficacy (% inhibition)
3	BID	0.074	0.702	30
10	BID	0.211	2.032	44
30	BID	0.716	6.909	66
100	BID	2.106	20.321	88
60	QD	0.026	6.909	13

Suppl Table S3. Modeled plasma exposure of JNJ-61803534 vs. observed efficacy in the CIA model

Suppl Figure S3

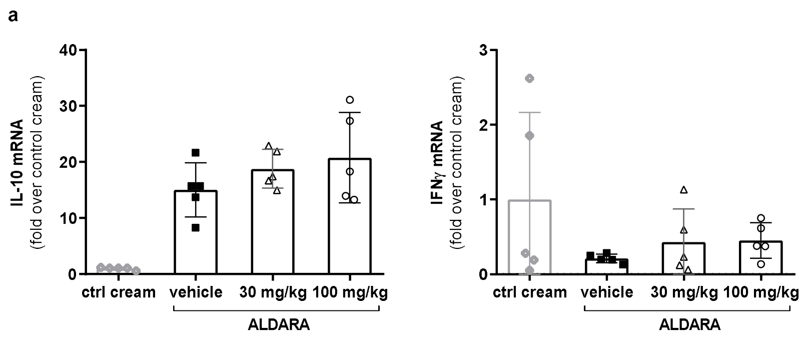

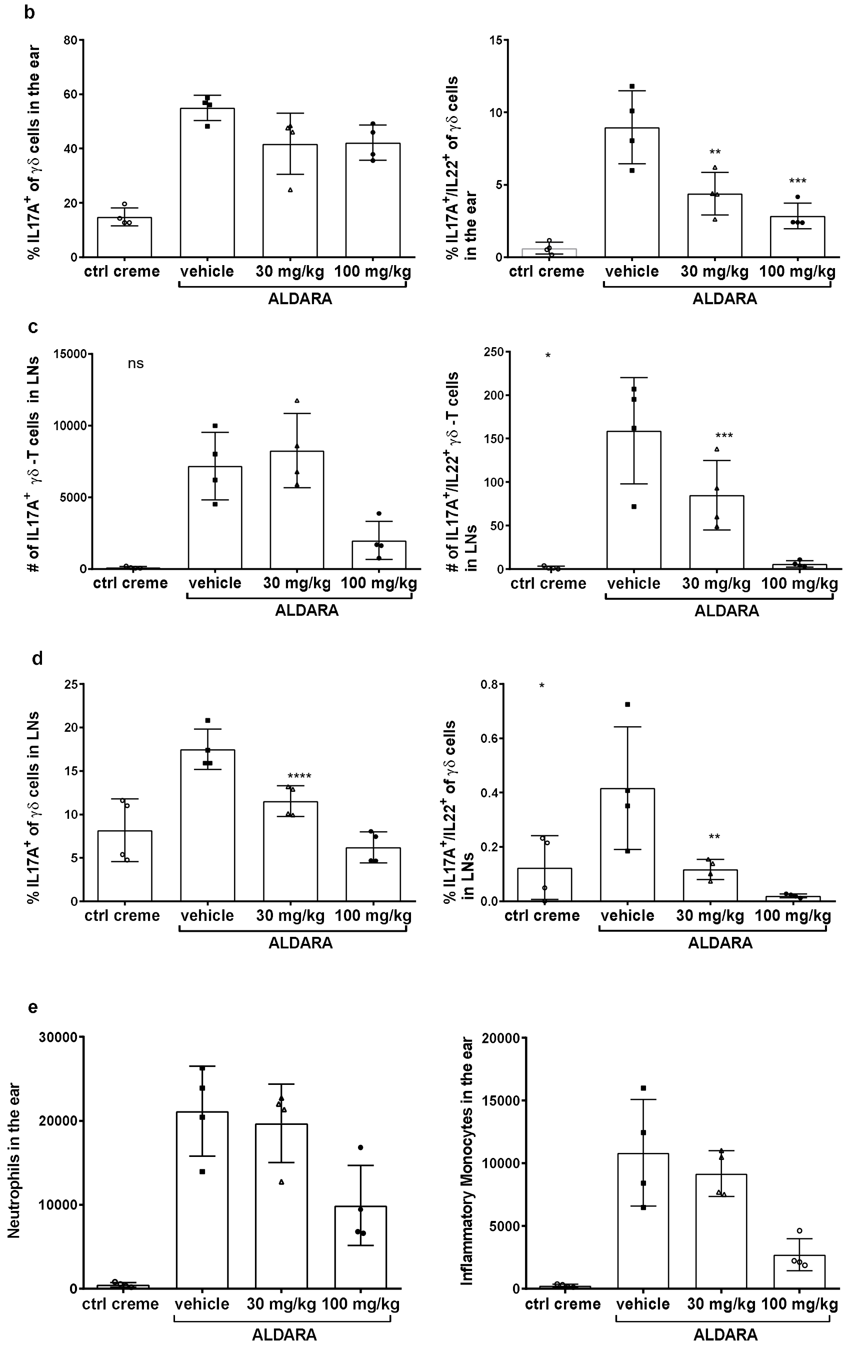

Suppl Figure S3. Effect of JNJ-61803534 in IMQ-induced skin inflammation in mice. (a) Gene expression (IL-10 and IFNγ) in the ears of the mice. Data are presented as mean ± SEM of fold change over control cream group for each individual animal (n=5). The relative expression level was calculated based on the formula: 2^ (β_2M CT- Target Gene CT) * 10,000. (b-d) Number and percentage of IL-17A$^+$ and IL-17A$^+$/IL-22$^+$ γδ T cells in the ears (b) and draining LNs (c-d), (e) Number of neutrophils and inflammatory monocytes in the ears. Data presented as mean ± SEM of 5 individual animal. Statistical analyses were performed with one-way ANOVA, *p<0.05, **p<0.01, ***p<0.001, and ****p<0.0001

Suppl Table S4

	Placebo n=12	10 mg n=6	30 mg n=6	100 mg (fasted) n=9	100 mg (fed) n=9	200 mg n=6
**Age, years**						
Mean (SD)	46.92 (10.423)	50.17 (13.227)	56.00 (2.366)	49.89 (10.799)	43.33 (13.124)	55.33 (5.317)
Median	49.00	53.50	56.00	56.00	49.00	57.00
Range	27.0 - 59.0	25.0 - 60.0	53.0 – 59.0	31.0 – 58.0	23.0 – 57.0	45.0 – 59.0
**Sex**						
Female	1	1	3	2	2	4
Male	11	5	3	7	7	2
**Race**						
American Indian or Alaska Native	0	0	0	0	1	0
White	12	6	6	9	8	6
Other	0	0	0	0	0	0
**Ethnic**						
Hispanic or Latino	1	0	0	0	0	0
Not Hispanic or Latino	11	6	6	9	9	6
**Weight, Kg**						
Mean (SD)	84.43 (14.551)	82.15 (10.284)	75.55 (12.177)	79.39 (6.098)	76.46 (9.330)	72.75 (8.745)
Median	86.05	81.30	79.25	77.80	73.90	71.90
Range	(55.2 - 112.3)	(72.4 - 100.4)	(55.3 - 89.2)	(70.2 - 90.8)	(62.3 - 91.1)	(60.4 - 86.2)
**Height, cm**						
Mean (SD)	179.50 (10.059)	178.67 (7.815)	175.00 (11.136)	174.56 (7.418)	176.33 (10.112)	167.50 (6.411)
Median	180.00	180.00	170.50	175.00	176.00	169.00
Range	(160.0 - 198.0)	(167.0 - 189.0)	(164.0 - 193.0)	(162.0 - 186.0)	(160.0 - 190.0)	(157.0 - 173.0)
**Body mass index, kg/m^2**						
Mean (SD)	26.07 (2.994)	25.75 (2.990)	24.62 (2.944)	26.06 (1.612)	24.53 (1.404)	26.02 (3.525)
Median	27.25	25.45	24.80	26.50	24.10	26.75
Range	(20.4 - 29.4)	(22.4 - 30.0)	(19.6 - 28.5)	(23.0 - 28.0)	(22.3 - 26.2)	(20.4 - 29.0)
Underweight <18.5	0	0	0	0	0	0
Normal 18.5-<25	4	3	3	2	5	3
Overweight 25-<30	8	2	3	7	4	3
Obese ≥30	0	1	0	0	0	0

Suppl Table S4. Summary of Demographics and Baseline Characteristics

Suppl Table S5

a

	**10 mg n=6**	**30 mg n=6**	**100 mg n=9**	**200 mg n=6**
**C_{max}, ng/mL**	35.9 (12.9)	146 (39.6)	700 (114)	1808 (357)
**t_{max}, h**	10.00 (6.00 – 24.00)	6.02 (6.00 – 8.00)	6.00 (4.00 – 8.02)	6.00 (2.00 - 6.02)
**AUC_{216h}, ng.h/mL**	5178 (1360)	17369 (4278)	68436 (14217)	153931 (46438)
**AUC_{last}, ng.h/mL**	-	-	99125 (24428)	200305 (66610)
**AUC_{∞}, ng.h/mL**	8476 (1494)	28679 (7938)	99976 (24827)	201067 (67162)
**$t_{1/2}$, h**	168.0 (49.4)	169.9 (70.0)	169.8 (22.6)	163.8 (18.0)
**CL/F, L/h**	1.20 (0.183)	1.11 (0.269)	1.06 (0.268)	1.10 (0.385)
**Vd_z/F, L**	288 (84.4)	270 (141)	254 (45.2)	253 (67.0)

b

	**100 mg, fasted n=9**	**100 mg, fed n=9**
**C_{max}, ng/mL**	700 (114)	602 (176)
**t_{max}, h**	6.00 (4.00 – 8.02)	12.02 (6.02 – 24.00)
**AUC_{216h}, ng.h/mL**	68436 (14217)	61701 (15085)
**AUC_{last}, ng.h/mL**	99125 (24428)	89708 (24389)
**AUC_{∞}, ng.h/mL**	99976 (24827)	90392 (24790)
**$t_{1/2}$, h**	169.8 (22.6)	168.3 (24.5)
**CL/F, L/h**	1.06 (0.268)	1.18 (0.303)
**Vd_z/F, L**	254 (45.2)	280 (61.2)

Suppl Table S5. Pharmacokinetic results of JNJ-61803534 after administration of a single dose in healthy volunteers. (a) under fasted condition; (b) under fasted and fed conditions. All presented as Mean (SD), except t_{max} as median (range). C_{max} = Maximum observed plasma concentrations during a dosing interval; T_{max} = Time to reach the maximum observed plasma concentration; AUC_{216h} = area under the plasma concentration-time curve from time 0 through 216 hours; AUC_{last} = Area under the plasma concentration-time curve from time 0 to time of the last quantifiable concentration; AUC_{∞}= Area under the plasma concentration-time curve from time 0 to infinite time, calculated as the sum of AUC_{last} and $C_{last/\lambda z}$, in which Clast is the last observed quantifiable concentrations; $t_{1/2}$ = Elimination half-life associated with the terminal slope (λz) of the semilogarithmic drug concentration-time curve, calculated as 0.693/λz; λz = First-order rate constant associated with the terminal portion of the curve, determined as the negative slope of the terminal log-linear phase of the drug concentration-time curve; CL/F = Total clearance of drug after extravascular administration, uncorrected for absolute bioavailability, calculated as: dose (D)/AUC; Vdz/F = Apparent volume of distribution after extravascular administration, uncorrected for absolute bioavailability.

Suppl Figure S4

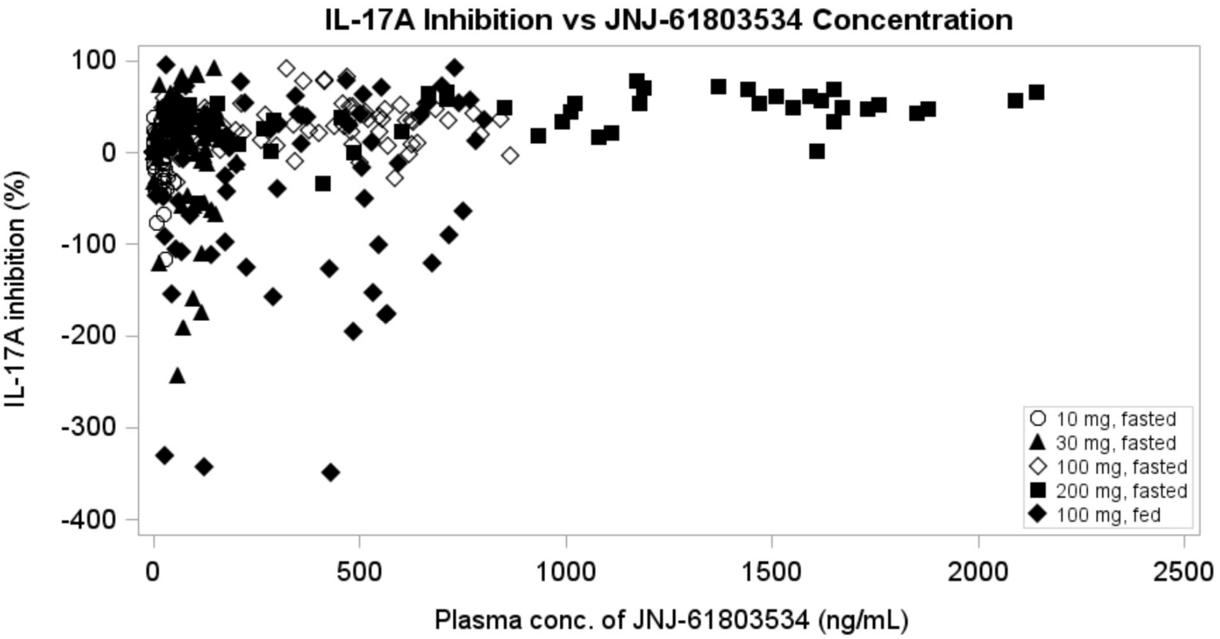

Suppl Figure S4. Scatter plot of IL-17A inhibition versus JNJ-61803534 plasma concentrations
